# HCV genotype 1a shows a better virological response to antiviral therapy than HCV genotype 1b

**DOI:** 10.1186/1471-230X-12-162

**Published:** 2012-11-16

**Authors:** Adriano M Pellicelli, Mario Romano, Tommaso Stroffolini, Ettore Mazzoni, Fabrizio Mecenate, Roberto Monarca, Antonio Picardi, Maria Elena Bonaventura, Cristina Mastropietro, Pascal Vignally, Arnaldo Andreoli, Massimo Marignani, Cecilia D’Ambrosio, Lucia Miglioresi, Lorenzo Nosotti, Olga Mitidieri, Umberto Vespasiani Gentilucci, Claudio Puoti, Giuseppe Barbaro, Angelo Barlattani, Caterina Furlan, Giorgio Barbarini

**Affiliations:** 1 Liver Unit Azienda Ospedaliera San Camillo Forlanini, Circonvallazione Gianicolense, 87 00149, Rome, Italy; 2 Liver Unit Ospedale Sandro Pertini, Via dei Monti Tiburtini 385, 00157, Rome, Italy; 3 Department of Infectious and Tropical Disease Policlinico Umberto I, Viale del Policlinico 155, 00161, Rome, Italy; 4 Liver Unit Policlinico Casilino, Via Casilina, 1049-00169, Rome, Italy; 5 Liver Unit Ospedale Villa Betania, Via Niccolò Piccolomini 27, 00165, Rome, Italy; 6 Infectious Disease Ospedale di Belcolle strada Sammartinese, 01100, Viterbo, Italy; 7 Liver Unit Campus Biomedico University, Via Álvaro del Portillo, 21 00128, Rome, Italy; 8 Infectious Disease Ospedale San Camillo de Lellis, Via John Fitzgerald Kennedy, 02100, Rieti, Italy; 9 Department of Infectious Disease Policlinico Umberto I, Viale del Policlinico 155, 00161, Rome, Italy; 10 National Institute of Health, Viale Regina Elena 299, 00161, Rome, Italy; 11 Department of Digestive and Liver Disease, Azienda Ospedaliera Sant'Andrea, Via Grottarossa, 1035/1039, Rome, Italy; 12 Medicine of Migration National Institute for Migrant Health and Poverty, Via di S. Gallicano 25/a, 00153, Rome, Italy; 13 Department of Internal Medicine and liver unit Ospedale Generale di Marino, Viale XXIV Maggio, 00047, Marino Rome, Italy; 14 Department of Medical Pathophysiology, University of Rome La Sapienza, Viale del Policlinico 155, 00161, Rome, Italy; 15 Liver Unit ASL RM/A, Rome, Italy; 16 Infectious and Parasitic Diseases, Policlinico San Matteo P.zzale Golgi,2, 27100, Pavia, Italy

**Keywords:** Genotype 1a, HCV genotype 1 subtypes, Sustained virological response, Antiviral therapy, Pegylated interferon

## Abstract

**Background:**

The impact of viral subtype on the rate of sustained virological response (SVR) to antiviral therapy in patients chronically infected with hepatitis C genotype 1 subtype 1a and 1b has not been extensively investigated. The aim of this study is to determine whether the HCV genotype 1 subtypes 1a and 1b respond differently to treatment with PEGylated interferon (PEG-IFN) plus ribavirin.

**Methods:**

For 48 weeks, 388 “naïve”genotype 1 patients were treated weekly with PEG-IFN α-2a or PEG-INF α-2b combined with daily ribavirin (1000–1200 mg/day). The numbers of patients in whom HCV-RNA was undetectable were compared after 4 (rapid virological response, RVR), 12 (early virological response, EVR), and 48 (end treatment virological response, ETR) weeks of treatment as well as 24 weeks after the last treatment (sustained virological response, SVR).

**Results:**

The rate of SVR was higher in subtype 1a patients than subtype 1b patients (55% vs. 43%; p < 0.02). Multiple logistic regression analysis showed that infection with genotype 1a (odds ratio(OR) : 1.8; 95% confidence interval (CI): 1.4 to 4.1), age < 50 years (OR:7.0; 95% CI 1.1 to 21.2), alanine aminotransferase level (ALT)<100 IU/ml (OR:2.1; 95% CI: 1.3 to3.5), HCV-RNA < 5.6 log_10_ IU/ml (OR: 3.2; 95% CI: 2.7 to 6.9) and fibrosis score < S3 (OR: 3.8; 95% CI:3.2 to 7.4), were all independent predictors of SVR.

**Conclusion:**

Dual antiviral therapy is more effective against HCV subtype 1a than against subtype 1b and this difference is independent of other factors that may favour viral clearance.

**Trial registration:**

ClinicalTrials.gov Identifier: NCT01342003

## Background

Despite the promise of new antiviral drugs that can act directly on hepatitis C viral replication such as protease and polymerase inhibitors, a 48 weeks course of PEGylated interferon (PEG-INF) combined with ribavirin remains the current standard treatment for genotype 1 chronic hepatitis C (CHC) [1,2]. Extensive research has shown that patients infected with HCV genotype 1 have a lower rate of viral response than those infected with genotype 2 or 3. In large randomized multinational trials, PEGylated interferon α-2a plus ribavirin has produced an SVR of about 50% in the more difficult-to-treat subgroup of patients infected with HCV genotype 1 [3,4]. Furthermore, advanced fibrosis is a predictor of non response to antiviral treatment in patients with genotype 1 virus [5-7]. Very few studies have examined whether the subtype of genotype 1(1a or 1b) affects the rate of SVR [7-10].

We have performed an observational study on a large cohort of “naïve” HCV patients to evaluate the influence of HCV subtypes 1 on the response to treatment with PEG-INF plus ribavirin.

## Methods

### Patients

A total of 11 regional centres affiliated with the CLEO Group partecipate in the study between February 2007 and October 2010. Eligible subjects were naïve patients infected with HCV genotype 1 virus who met the internationally recognised criteria for treatment (elevation of aminotransferases and inflammation and/ or fibrosis at liver biopsy). The exclusion criteria included co-infection with human immunodeficiency virus (HIV) or hepatitis B virus (HBV), alcohol intake averaging greater than 20 g per day, active drug abuse, chronic systemic disease, psychiatric disorders, autoimmune disease, pregnancy or lactation. The following data were collected: age, gender, body mass index (BMI) and Ishak score of liver biopsy [11]. Of the 388 patients, 322 provided informed consent for liver biopsy.

All patients received Peg-IFN α-2a at 180 μg/week or PEG-INF α-2b at 1.5 μg/kg/week combined with ribavirin at 1000 mg/day if the body weight was < 75 Kg or 1200 mg/day if the body weight was > 75 kg. The dose of PEG-INF and ribavirin were modified as necessary according to the standard criteria and protocol [12]. Patients with undetectable HCVRNA at week 4 were considered rapid virological responders (RVR) and were treated for full 48 weeks. Patients with a < 2 log decline in HCVRNA at week 12 or who remained HCVRNA positive at week 24 were considered to be non-responders and did not continue with the treatment regimen. All patients who withdrew from the study were also defined as non-responders. The primary end point was sustained undetectable serum HCVRNA 24 weeks after the end of treatmen (SVR).

### HCVRNA quantification

Quantitative determination of HCVRNA (TaqMan Roche Diagnostics). was performed before the treatment. The TaqMan value used to determine the response was 15 IU/ml. The TaqMan method is a standardised technique that was used in all the CLEO group centres beginning in December 2007. HCVRNA level was expressed as log_10_ IU/ml. HCVRNA was measured before the treatment, at weeks 4,12,24,48 of treatment and 24 weeks after the final treatment. HCV genotyping was performed using a hybridisation technique (INNOLiPA HCV Immunogenetics).

### Statistical analysis

All analysis were performed on the basis of the intention to treat (ITT);i.e., the denominator included all subjects who received at least 1 dose of treatment.

Statistical analysis was performed using the Epiinfo software package. All Data were expressed as the median and range for discrete variables and as counts and percentages for qualitative variables. The differences between the groups were compared using non parametric tests (the Mann Whitney U test for continuous variables and χ^2^ test for parametric variables). A p value of < 0.05 was considered to be significant.

The crude odds ratios (O.R s) for the association of SVR with different variables were evaluated by univariate analysis. The following variables were analyzed: sex, age (cut-off 50 years), ALT (cut-off 100 IU/ml), gamma-glutamyltranspeptidase (GGT), BMI (cut-off 24.9), HCV-RNA (cut-off 400,000 IU/ml), HCV genotype 1 subtype ( 1a or 1b), liver biopsy grade and stage score, and type of PEGylated interferon received. The adjusted O.R. were calculated by multiple logistic regression analysis in order to identify independent predictors of SVR. Adjustment were made for all of the variables considered at univariate analyses.

### Ethics

The study was approved by a central ethic committee (San Camillo Hospital Rome Italy).

## Results

The study included 388 patients; of these 165 were infected with HCV genotype 1 subtype 1a (42.5%) and 223 with subtype 1b (57.5%). On average, subtype 1a patients were younger and had lower baseline HCV-RNA levels than subtype 1b patients. All the baseline characteristics of the 388 patients are reported in Table 1.

**Table 1:** Baseline Characteristics of study population according to HCV subtype

	**Genotype 1a (n = 165)**	**Genotype 1b (n = 223)**	**P value**
Sex (M/F)	120/45	123/100	0.001
Age (y)	45.0 ± 10.6	49.0 ± 12.1	0.0001
BMI (kg/m^2^)	24.1 ± 3.2	24.0 ± 3.3	ns
ALT (IU/ml)	109.2 ± 68.7	101.4 ± 61.2	ns
GGT (IU/ml)	65.1 ± 39.1)	67.3 ± 54.5	ns
Hgb (gr/dL)	15.2 ± 1.3	14.8 ± 1.9	ns
HCV-RNA ( log_10_ IU/ml)	5.72 ± 0.7	5.9 ± 0.5	0.006
HCV-RNA n (%)			
≤ 5 .60 log_10_	52 (32)	44 (20)	0.01
> 5 .60 log_10_	113 (68)	179 (80)	0.01
Staging n (%)			
≤ S3	111 (85)	151 (79)	ns
> S3	19 (15)	41 (21)	ns
Source of Infection n (%)			
-BT	69 (41)	85 (38)	ns
-DA	78 (47)	92 (41)	ns
-S	8 (5)	15 (7)	ns
-UN	11 (7)	32 (14)	0.03

Legend: M: male, F: female, y:years, BMI: body mass index, ALT: alanine aminotransferase value, GGT: gamma-glutamiltranspeptidasi, Hgb:Haemoglobin value, BT: blood trasfusione, DA:drug abuse, S: sexual, UN: unknown.

### Virological response

**Figure 1:**
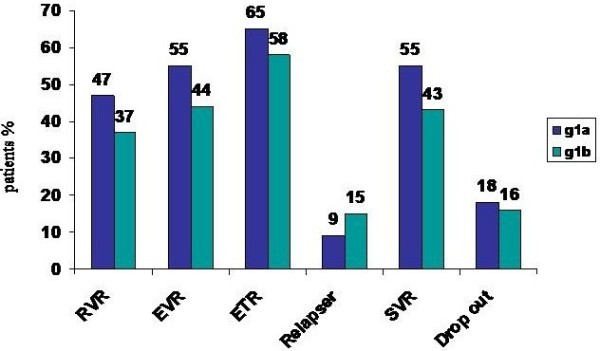
Rapid virological response (RVR), early virological response (EVR), end treatment virological response (ETR), sustained virological response (SVR) and drop out in genotype 1 subtypes 1a and 1b patients.

At week 4 of treatment HCV-RNA was undetectable (RVR) in 77 genotype 1a patients (47%) and in 83 genotype 1b patients (37%) ( p < 0.07). At week 12 of treatment 91 genotype 1a patients (55%) and 99 genotype 1b patients (44%) had undetectable HCV-RNA (early virological response; EVR) (p < 0.04). At the end of treatment 108 genotype 1a patients (65%) and 131 genotype 1b patients (58%) had undetectable HCV-RNA (p = n.s). Seventeen (10%) of the genotype 1a patients and 32 (14%) of the genotype 1b had detectable HCVRNA at week 12 but not at week 24 (slow responders) (p = ns).At the end of the treatment, there were 16 (9%) relapsed patients in the genotype 1a group and 35(15%) in the genotype 1b group (p = ns). Sustained virological response was attained in 91 genotype 1a patients (55%) and 96 genotype 1b patients (43%) ( p < 0.02). A total of 18 genotype 1a and 16 genotype 1b patients discontinued all treatments at some time during the study owing due adverse events (Figure 1).

### Sustained virological response

The overall rate of SVR was 48.2%. Subtype 1a, age < 50 years, ALT value < 100 IU/ml, HCV-RNA < 400.000IU/ml (< 5 .60 log_10_ IU/ml), and fibrosis score ≤ S3 were all factors predisposing to SVR (Table 2). The rate of SVR in patients with fibrosis score S0-S3 was significantly higher for subtype 1a (62%) than for subtype 1b (48%) ( p < 0.03); while no statistically significant difference in SVR was observed between the 2 subtypes (1a and 1b) in patients scoring S4-S6 (31.5% vs. 28%; p = n.s.).(Data not shown).

**Table 2:** Overall SVR according to different variables

**Variable**	**N° of pts/Tot pts**	**SVR (%)**	**P value**
Sex			
F	68/145	46	ns
M	119/243	49	
Age			
< 50	123/210	58	0.00001
> 50	64/178	37	
BMI			
< 25	145/280	51	0.05
> 25	32/82	39	
ALT			
< 100	139/261	53	0.002
> 100	51/127	40	
HCV-RNA			
≤ 5 .60 log_10_	64/96	66	0.00004
> 5 .60 log_10_	123/292	42	
Genotype			
1a	91/165	55	0.02
1b	96/223	43	
Staging*			
≤ S3	142/262	54	0.00008
> S3	15/60	25	
Drug			
PegINF α 2b	100/195	51	ns
PegINF α 2a	87/193	45	
RVR			
Yes	129/160	80	0.000001
No	58/228	25	

Legend. SVR: sustained virological response, F: female, M:male, PegINF: pegylated interferon, BMI: body mass index, ALT: alanine aminotransferase value (IU/ml); RVR: rapid virological response.

*322 over 388 patients were submitted to liver biopsy and analyzed for this variable.

Table 3 shows the crude and adjusted Odds Ratios (ORs) for the associations of different variables with SVR. After adjustment for the influence of the confounding variables by logistic regression analysis, age < 50 (OR:7.0; 95% CI: 1.1 to 21.1), genotype 1a (OR: 1.8; 95% CI: 1.4 to 4.1), HCV-RNA < 5.6 log_10_ IU/ml (OR: 3.2; 95% CI: 2.7 to 6.9), a fibrosis score ≤ S3 (OR: 3.8; 95% CI:3.2 to 7.4;), and ALT value < 100 IU/ml (OR:2.1 95% CI: 1.3 to 3.5) were all independent predictors of SVR while sex, and BMI were not associated with SVR.

**Table 3:** Factors associated with the likelihood of SVR. Crude and adjusted Odds Ratios (O.R.) derived by multiple logistic regression analysis

	**Crude O.R. (95% CI)**	**Adjusted O.R.**	**(C.I. 95%)**
HCV-RNA ≤ 5.6 log_10_ IU/ml	2.9 (1.8-5.1)	3.2	(2.7-6.9)
≤ S3*	3.8 (2.0-7.3)	3.8	(3.2-7.4)
Subtype 1a	1.9 (1.2-2.9)	1.8	(1.4-4.1)
Age	7.4 (0.9-40.0)	7.0	(1.1-21.2)
< 50			
> 50			
BMI	1.7 (1.0-2.8)	1.4	(0.8-2.5)
< 25			
> 25			
ALT	1.9 (1.2-3.0)	2.1	(1.3-3.5)
< 100			
> 100			
Sex	1.1 (0.7-1.7)	1.3	(0.8-2.2)
RVR	21.0 (11.5-38.3)	3.2	(1.3-7.7)

Legend O:R: odds ratio; BMI: body mass index, ALT: alanine aminotransferase value IU/ml RVR rapid virological response.

*322over 388 patients were submitted to liver biopsy and analyzed for this variable.

Cross-tabulation of RVR vs.SVR showed that the positive predictive value (PPV) of RVR for the achievement of SVR was 82.0% for subtype 1a and 77.4% for subtype 1b (data not shown).

### Safety profile

Eighteen subtype 1a (11%) and 16 subtype 1b (7%) patients stopped antiviral treatment due to adverse effects. Six patients stopped during the first month of treatment and the remaining patients during the following months. The percentage of patients in both groups whose treatment dose were decreased due to adverse events were comparable: 9.6% (16 patients) for subtype 1a and 10.7% (24 patients) for subtype 1b. Anaemia was the most frequent cause of dose reduction. Total discontinuation, dose modifications and adverse events related to treatment are showed in Table 4.

**Table 4:** Total discontinuation, dose modification and adverse events related to antiviral treatment

	**Subtype 1a n = 165**	**Subtype 1b n = 223**
Total discontinuation n (%)	18 (11)	16 (7.1)
-depression	7 (4.2)	3 (1.3)
-fatigue	10 (6)	9 (4)
-hyperthiroidism	0	1 (0.4)
-anemia	1 (0.6)	3 (1.3)
Adverse events n (%)	7 (4.2)	12 (5.3)
-depression	1 (0.6)	3 (1.3)
-hypothiroidism	1 (0.6)	0
-anemia	5 (3.0)	7 (3.1)
-neutropenia	0	2 (0.9)
Dose modification n (%)	16 (9.6)	24 (10.7)
Peginterferon	9 (5.4)	11 (5)
Ribavirin	7 (4.2)	13 (5.8)

n:number.

## Discussion

We have conducted a large observational study to assess the influence of viral subtype within HCV genotype 1 on the virological response to antiviral treatment in naïve HCV patients. Logistic regression analysis showed that HCV subtype 1a, mild liver fibrosis scored as less than S3 (Ishak score), HCV-RNA level less than 5.6 log_10_ IU/ml, age less than 50 years, and ALT level less than 100 IU/ml were all independent predictors of SVR.

Many efforts have been made to identify predictors of SVR to antiviral treatment in the difficult-to- treat chronic hepatitis C genotype 1 patients. Liver histology and viral HCV-RNA levels seem to be particularly important predictor of response in these patients. A recent study by Cheng et al. showed that naïve genotype-1 patients with advanced fibrosis were less likely to achieve SVR than those without advanced fibrosis [5]. Bruno et al. demonstrated that age and liver fibrosis predicted the response rate to PEG-INF and ribavirin combination therapy [6]. Few studies have investigated the impact of viral subtype on SVR genotype 1 patients. A study by Legrand-Abravanel et al. showed by multivariate analysis that genotype 1 subtype 1a was associated with a lower response to HCV therapy than subtype 1b [8]. However, this was an observational study with some potential confunding factors: more than 23% of the patients were concomitantly infected with HIV or HBV; and nearly 35% were interferon experienced patients. Similarly, Nicot et al. found that genotype 1b and HCVRNA < 15IU/ml were the only independent predictors of SVR in genotype 1 patients. However the population of patients analysed in this study was not homogeneous: 23% of all patients were coinfected with HIV and 42% had not responded to previous interferon treatment [9]. Zein et al. found no difference in SVR rates between subtype 1a and 1b patients treated with standard interferon [13]. The PROBE study, which included more than 6000 HCV infected patients, showed that SVR was marginally associated with subtype 1a ( OR 1.41; 95% CI 1.0-2.03) [7]. A recent observational study with a retrospective and prospective phase conducted in Italy (AIFA study), which included naïve, relapser and no responder patients, showed that genotype 1a naive patients experienced a rate of SVR around 6% higher than that observed for genotype 1b naïve patients and comparable to that observed in genotype 4 (retrospective phase-SVR G1a versus G1b 37.1 vs 31.6% p < 0.001 and prospective phase-SVR 31.0 vs 26.5% p < 0.001) [10]. A higher rate of adverse events and in particular of anemia reported in AIFA study, could have influenced the different rate of SVR observed respect to our study. We hypothesize that, in particular, an high rate of anemia could have been responsible for ribavirin or peginterferon dose modification in the AIFA study (data not reported).

Genotype 1 subtypes 1a and 1b are the most common HCV genotypes in the United State. These subtypes are also predominant in Europe and subtype 1b is responsible for up to 73% of HCV infection in Japan. Zein et al. found that patients with HCV subtype 1b were older on average than those infected with other genotypes and that subtype 1b may have been present in some countries before the other genotypes. All patients who acquired HCV before 1955 were infected with subtype 1b. Subtype 1a was introduced in the late 1950s and then, it became the most prevalent genotype [13]. According to this model HCV subtype 1b is associated with more severe liver disease not because it is a more aggressive form of HCV but because it reflect a longer duration of infection [14]. In our study more genotype 1b than genotype 1a patients relapsed after treatment although the difference was not statistically significance. This difference could have been due to the higher percentage of slow responding patients in genotype 1b group than in genotype 1a group. Some studies have demonstrated a significant association between slow response and relapse in patients with an EVR [15,16]. Although genotype 1a present a lower age and lower baseline HCVRNA level respect to genotype 1b patients, the logistic regression analysis and in particular Adjusted odds ratio shows the independent influence of genotype 1a on SVR without the disturbing influence of other variables. For the above- mentioned reasons subtype 1b patients may respond less favourably than subtype 1a patients to PEG-INF plus ribavirin.

While we observed in dual antiviral therapy a better SVR of genotype 1a respect to genotype 1b patients, genotype 1a presents higher virologic failure respect to genotype 1b in patients treated with triple antiviral combination therapy including protease inhibitors Boceprevir or Telaprevir. Overall, the barrier to resistance is lower in genotype 1a than in genotype 1b strains, resulting in higher breakthrough rates in the former [17].

In our study we found similar SVR in patients treated with pegylated interferon alfa-2a and alfa 2-b, this is in agreement with Ideal and AIFA study [10,18] but it is in contrast with two italian randomized controlled studies that demonstrated higher SVR in genotype 1 patients treated with pegylated interferon alfa-2a [19,20]. At present the superiority of one regimen over the other in terms of treatment efficacy remains unknown. The performance of the two drugs has not been explored in patients stratified by treatment modifiers such as fibrosis stage, basal viral load, insulin resistance, age and it is unlikely that future effort will extend current knowledge as we enter in the era of protease and polymerase inhibitors [21].

The interleukin-28B (IL28B) polymorphism has been reported to influence viral kinetics and SVR in genotype 1 patients [22]. We did not determine this parameter in the present study; its significance was not known at the time that the study was conducted. Therefore, we cannot exclude the possibility that our subtype 1a patients may have had a more favourable IL28B polymorphism profile than did the subtype 1b patients. IL28B polymorphism could be an additional parameter explaining the uniquely higher SVR rate for subtype 1a versus 1b observed in the Italian population.

Finally, we would like to emphasise that this study was conducted in “real- world- patients”, thus providing a representative picture of HCV treatment.

## Conclusion

While in triple antiviral combination therapy including protease inhibitors Boceprevir or Telaprevir genotype 1a presents higher virologic failure respect to genotype 1b patients, we observed in dual antiviral therapy a better SVR of genotype 1a respect to genotype 1b patients that is independent of other factors that may favour viral clearance.

## Competing interests

All the authors declares the they not have received reimbursements, fees, funding, or salary from an organization that may in any way gain or lose financially from the publication of this manuscript, either now or in the future. All the authors declares that they do not hold any stocks or shares in an organization that may in any way gain or lose financially from the publication of this manuscript, either now or in the future. All the authors declare that they do not hold or apply any patents relating to the content of the manuscript. All the authors declare that they have not received reimbursements, fees, funding, or salary from an organization that holds or has applied for patents relating to the content of the manuscript. All the Authors declare that they not have any other financial competing interests.

## Authors’ contributions

AMP Concept-Design-Manuscript editing-Manuscript review-Manuscript preparation, Data acquisition MR Concept-Design-Literature search manuscript review, manuscript preparation, TS Statistical analysis, manuscript editing, manuscript review. LN Data acquisition, literature search. FM Data acquisition, manuscript review. CP Data acquisition, manuscript preparation, analysis of laboratory data. GB Statistical analysis ,Manuscript review. RM Data acquisition and manuscript review. EM Manuscript editing, Data acquisition. AP Data acquisition. MEB Manuscript review, data acquisition. CM Data acquisition. PV statistical analysis, data acquisition, manuscript editing. AA Manuscript editing, literature search. MM Literature search, data acquisition. CD Manuscript preparation, Data acquisition, Concept. LM Manuscript review, Data acquisition. OM Laboratory analysis, Data acquisition. UVG data acquisition. Manuscript preparation. CF Manuscript review and preparation, critical review of the mauscript AB Manuscript preparation, Data acquisition. GB Data acquisition, Manuscript preparation-Literature search, Manuscript review. All the authors read and approved the final manuscript

## Pre-publication history

The pre-publication history for this paper can be accessed here:

http://www.biomedcentral.com/1471-230X/12/162/prepub
